# The Hormetic Effect of Metformin: “Less Is More”?

**DOI:** 10.3390/ijms22126297

**Published:** 2021-06-11

**Authors:** Isabella Panfoli, Alessandra Puddu, Nadia Bertola, Silvia Ravera, Davide Maggi

**Affiliations:** 1 Biochemistry Lab., Department of Pharmacy, University of Genoa, Viale Benedetto XV, 3, 16132 Genoa, Italy; Isabella.Panfoli@unige.it; 2 Department of Internal Medicine and Medical Specialties, University of Genoa, Viale Benedetto XV, 6, 16132 Genoa, Italy; alep100@hotmail.com; 3 Department of Experimental Medicine, University of Genoa, Via De Toni, 14, 16132 Genoa, Italy; nadia.bertola@gmail.com

**Keywords:** adipose tissue, diabetes, gut, endothelium, heart, hormetic effect, liver, MTF, skeletal muscle

## Abstract

Metformin (MTF) is the first-line therapy for type 2 diabetes (T2DM). The euglycemic effect of MTF is due to the inhibition of hepatic glucose production. Literature reports that the principal molecular mechanism of MTF is the activation of 5′-AMP-activated protein kinase (AMPK) due to the decrement of ATP intracellular content consequent to the inhibition of Complex I, although this effect is obtained only at millimolar concentrations. Conversely, micromolar MTF seems to activate the mitochondrial electron transport chain, increasing ATP production and limiting oxidative stress. This evidence sustains the idea that MTF exerts a hormetic effect based on its concentration in the target tissue. Therefore, in this review we describe the effects of MTF on T2DM on the principal target organs, such as liver, gut, adipose tissue, endothelium, heart, and skeletal muscle. In particular, data indicate that all organs, except the gut, accumulate MTF in the micromolar range when administered in therapeutic doses, unmasking molecular mechanisms that do not depend on Complex I inhibition.

## 1. Introduction

Type 2 diabetes mellitus (T2DM) will affect 500 million people worldwide by 2030 [1]. Metformin (MTF; 1,1-dimethyl biguanide) is an oral drug employed to reduce blood glucose concentration, and the current first-line pharmacological treatment for T2DM [2,3]. The mechanistic events linking MTF to its plasma glucose-lowering effect remain incompletely understood. For example, several authors have proposed that MTF exerts its primary antidiabetic action through the suppression of hepatic glucose production [4,5] in T2DM patients, inhibiting, by several mechanisms, hepatic gluconeogenesis. Others suggest that MTF induces the inhibition of transepithelial glucose transport in the intestine, lowering blood glucose levels during the early response to oral administration of MTF [6]. An alternative hypothesis by Madiraju et al. (2014) suggests that a change in cellular redox potential, not energy charge, explains the effect of MTF [7]. Moreover, the safety of MTF greatly increased its association with all hypoglycemic drugs [8,9]. MTF exerts pleiotropic effects, probably independent of glycemia regulation, such as end-organ injury and mortality reduction in patients with autoimmune diseases [10] and the modulation of miRNA expression in extracellular vesicles in T2DM patients [11]. MTF seems also involved in the reduction of neurodegenerative disease symptoms [12], although, in this case, a vitamin B12 supplementation should be associated [13].

MTF displays a high hydrophilicity that limits its permeability through lipid membranes [14]. Therefore, it is internalized in the cell by several transporters, including Organic Cation Transporters (OCTs) and multidrug and toxin extrusion (MATE) transporters expressed on the membrane surface [15,16]. Moreover, MTF is not metabolized and is excreted intact by the kidney and the gut [7].

The apparent volume of distribution (Vd) of MTF is a largely debated topic. Some authors reported a value between 63 and 267 L [17,18], while other authors indicated higher values [18] (300 L equal to 4.28 L/kg). These apparent discrepancies could depend on the method of drug administration, the dosage, and the duration of therapy. Indeed, this debate is at the heart of the issue of understanding the effects of MTF on various tissues. In detail, it has been proposed that most of the effects of MTF millimolar concentrations depend on its specific inhibition of the mitochondrial respiratory chain Complex I [19]. The consequences of Complex I inhibition are ATP production lowering and increase in AMP, in turn promoting the 5′-AMP-activated protein kinase (AMPK) activation and decrease the NAD^+^/NADH ratio [20,21]. Notably, some effects of MTF can be mimicked by Complex I inhibitors [21,22]. Inhibition occurs preferentially when the enzyme is in its catalytically incompetent “deactive” conformation; therefore, MTF should be added prior to catalysis initiation [23]. On the other hand, a similar mechanism has also been described for the MTF-induced inhibition of hexokinase [24]. Interestingly, the inhibition of tumor growth by MTF was hampered in cells expressing a MTF-resistant yeast analogue of Complex I (NDI1), suggesting that inhibition of Complex I is an important mechanism of the action of MTF, also against cancer [21,25]; in fact, MTF was shown to reduce the aerobic ATP production in many cancer cells [26], although its effect seems to depend on tumor adaptive strategies, cancer stem cell selection, tumors metabolism, and microenvironment [27].

However, at therapeutic doses, the plasma MTF concentration is in the micromolar range [7,28]; therefore, to cause Complex I inhibition, it has been hypothesized to accumulate in mitochondria, thanks to the mitochondrial transmembrane electrochemical potential. On the other hand, a high concentration of the positively charged MTF molecules would collapse the inner membrane potential [29]. Moreover, MTF is a hydrophilic compound, and may not cross the inner mitochondrial membrane without a specific transporter, not yet identified [19]. Thus, some studies suggest an indirect action of MTF on Complex I [21]. Indeed, MTF did not accumulate in liver mitochondria of rats treated orally with [^14^C] MTF [30].

**Figure 1:**
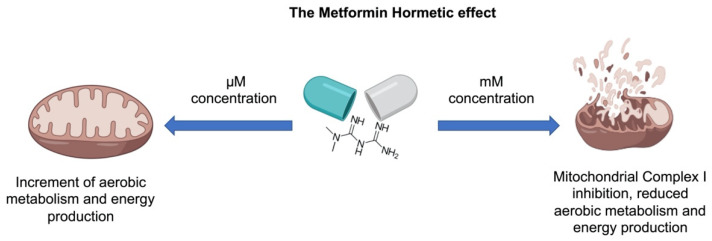
The metformin hormetic effect. Based on concentration, MTF exerts opposite effects. At µM concentrations, MTF increases the oxidative phosphorylation activity, enhancing aerobic energy metabolism. At mM concentrations, MTF inhibits the Complex I activity, reducing the aerobic energy production.

Notably, while millimolar MTF concentrations are needed to inhibit isolated Complex I, micromolar concentrations of the drug activate the mitochondrial aerobic metabolism, including the Complex I activity. For example, it was observed in Fanconi Anemia cells that micromolar MTF concentrations reactivate the mitochondrial respiration; conversely, millimolar concentrations induced the complete inhibition of Complex I [31]. Moreover, other studies have demonstrated that MTF increased respiration [32,33] and ATP content in human [34], mice [19], and bovine [35] samples. In other words, MTF displays a hormetic mechanism, exerting opposite effects depending on the concentration [31,35]: micromolar amounts, similar to that employed in in vivo treatments, increase the mitochondrial aerobic metabolism; by contrast, millimolar concentrations reduce the respiratory Complex I activity, causing a decrement of cellular energy availability (Figure 1). Moreover, MTF seems to act as a modulator of other mitochondrial functions, such as mitochondrial permeability transition pore (mPTP) opening, reactive oxygen species (ROS) production, and calcium storage. For example, MTF used at micromolar concentration inhibits the mPTP opening by an AKT-dependent pathway [36], preventing the associated ROS generation and the release of cytochrome c [37]. Conversely, MTF millimolar concentration determines mitochondrial swelling due to the endoplasmic reticulum (ER) stress and subsequent calcium uptake into the mitochondria [38]. However, the same authors described the inhibition of mPTP that, in this case, seems to be associated with the inhibition of Complex I activity [38]. On the other hand, the diabetic systemic milieu also influences the mitochondrial processes, such as energy production, ROS generation, calcium metabolism, and apoptosis regulation [39,40], negatively influencing hepatocyte metabolism, cardiac output, skeletal muscle contraction, beta-cell insulin production, and neuronal health [39].

Therefore, to evaluate the metabolic effect of MTF, it is necessary to consider the amount and activity of plasma membrane transporters as well as the plasma and tissue MTF concentrations and the relative volume occupied by the mitochondria inside the cell.

## 2. The Effects of Metformin (MTF) on Its Main Target Organs

### 2.1. The Effect of MTF on the Gut

After oral administration, ∼50% of MTF is absorbed through the upper small intestine and delivered to the liver. The remaining ∼50% unabsorbed MTF accumulates in the gut mucosa of the distal small intestine, reaching 30–300-fold of its concentration in plasma and the other tissues [41,42], thus rendering the intestine the main MTF reservoir, due to high absorption and accumulation.

It has been proposed that the high accumulation of MTF in enterocytes may be closely related to its gastrointestinal (GI) side effects [43,44,45,46]. This hypothesis is also supported by evidence that the use of modified formulations of MTF, which allow a gradual absorption, increase its tolerability [47,48,49]. Interestingly, some of the mechanisms involved in mediating the glucose-lowering effects of MTF are also responsible for its side effects, thus creating a fine metabolic balance that links the improvement of glycemic compensation to the side effects. MTF is absorbed into the enterocytes through cationic transporters with a saturable and dose-dependent process [41]: the plasma membrane monoamine transporter (PMAT) and organic cation transporter 3 (OCT3), localized on the luminal side of enterocytes [50], and, to a lesser extent, by the serotonin transporter (SERT) and carnitine/organic cation transporter OCTN1/SLC22A4, which are localized on the apical membrane of enterocytes [51,52]. Then, MTF is released into the portal vein mainly through organic cation transporter 1 (OCT1), which has been found in both apical and basolateral membrane of enterocytes [53,54,55]. In vitro studies showed that the efflux of MTF is less efficient in comparison with the uptake [41], suggesting that this imbalance may explain the higher concentrations in the intestine compared to other tissues. Therefore, especially the small intestine may be the only tissue in which MTF may reach a sufficient concentration to inhibit Complex I activity. This concept is supported by the increment of plasma lactate after MTF administration, which appears to derive from the intestine and not from the muscle, suggesting a metabolic switch to the anaerobic glycolysis due to the inhibition of Complex I [56].

Activation of AMPK by MTF is responsible for the inhibition of the nuclear bile acid receptor farnesoid X receptor (FXR) [57]. FXR is involved in ileal absorption of bile acids; therefore, its inhibition results in reduced bile acid absorption and consequent increased excretion in T2DM patients treated with MTF [58]. This pharmacological effect may cause gastrointestinal disturbances by altering the microbiome and stool consistency. On the other hand, the elevated fecal excretion of bile salts during MTF treatment is associated with metabolic improvement due to reduced circulating cholesterol and increased secretion of the Glucagon-Like Peptide 1 (GLP-1), a gut hormone produced by enteroendocrine L cells in response to nutrient ingestion [59].

It has been shown that a rise in GLP-1 secretion by MTF treatment may contribute to its glucose-lowering effects [60,61]. GLP-1 is involved in maintaining glucose homeostasis at various levels: it stimulates glucose-induced insulin secretion, inhibits glucagon secretion, gastric emptying, and food intake [62]. However, once released, it is rapidly inactivated by dipeptidyl peptidase 4 (DPP-4). The mechanism through which MTF increases GLP-1 concentration is not well known, but several studies indicated that MTF acts by increasing GLP-1 secretion rather than preventing its degradation [63,64,65,66]. Firstly, MTF increases glucose retention in the lumen of intestine [67]; therefore, GLP-1 secretion may be stimulated by the rise in glucose concentration [4]. Furthermore, as described above, MTF may induce GLP-1 secretion through elevation of bile acids. Indeed, several studies report that bile acids directly induce GLP-1 secretion through activation of the G protein–coupled bile acid receptor GPBAR1 (TGR5) [63,64,65]. It has also been hypothesized that MTF activation of AMPK may be another mechanism through which MTF increases the secretion of GLP-1. Indeed, MTF-induced GLP-1 secretion is blocked by pharmacological inhibition of AMPK activity [60,61]. However, considering that MTF did not increase GLP-1 secretion, and that activation of AMPK reduced GLP-1 secretion in in vitro models of L cells [66,68,69], it is conceivable that the rise in GLP-1 may be due to indirect effects of MTF on the gut. Finally, MTF may increase GLP-1 secretion through modulation of the gut microbiota, especially improving Short Chain Fatty Acids (SCFAs production) [70,71].

MTF contributes to glucose lowering by increasing glucose uptake and utilization in the intestinal tissue. These effects are probably achieved through regulation of the expression and distribution of glucose transporters, such as Glucose transporter 2 (GLUT2) and Sodium/glucose cotransporter 1 (SGLT1) in enterocytes. However, the relative contribution of glucose transporters is still debated. GLUT2 is localized mainly in the basolateral membrane of enterocytes, but in the presence of high glucose concentrations, it is rapidly translocated to the brush border membrane [72,73]; by contrast, SGLT1 is localized in brush border membrane and intracellular vesicles of enterocytes [74,75]. Sakar et al. demonstrated that MTF counteracts the abundance of SGLT-1 induced by glucose, and at the same time, increases GLUT2 protein levels in the brush border membrane of enterocytes [76]. On the contrary, Yang et al. showed that MTF increases the GLUT-dependent glucose uptake by increasing GLUT1 expression, with concomitant reduction of both GLUT2 and SGLT1 [77]. Recently, it has been shown that MTF also controls glucose homeostasis by inhibiting glucose transport from intestinal lumen to circulation [6]. In addition, Yang et al. showed that MTF inhibition of the electron transport chain results in an increase of GDF-15, a stress response cytokine which reduces appetite [77,78], from intestinal cells [77,79]. Reduced appetite can consequently limit the glucose intake, improving glycemic control. The increment in glucose uptake coupled with anaerobic glucose utilization induced by MTF leads to an increase in lactate production in enterocytes [67,80]. Furthermore, MTF may also contribute to the rise in lactate by inhibiting bacterial glycerophosphate dehydrogenase [81], and favoring the proliferation of lactate-producing bacteria, which may, in turn, be responsible of some or all the GI side effects of MTF [82,83,84]. However, evidence indicates that lactate may contribute to the beneficial effects of MTF [85]. For instance, lactate is mainly fermented to butyrate by the gut microbiota [86], and it is conceivable that butyrate may contribute to the glucose-lowering effects of MTF by stimulating incretin release.

The gut microbiota is altered in T2DM, and treatment with MTF leads to changes in bacterial composition [87,88,89]. Indeed, the amelioration of glycemic control and bile acid metabolism in MTF-treated T2DM patients correlate to changes in the microbiota composition, especially of *Firmicutes* and *Bacteroidetes* [90]. Evidence suggests that modulation of the gut microbiota might be involved in the glucose-lowering effects of MTF [4,70]. However, MTF-induced GI side effects has also been related to alterations in the microbiome. Considering that disturbances of GI occur soon after treatment [91], they probably cannot be attributed to a change in the microbiota composition, but rather to its inter-individual composition. Interestingly, GI intolerance decreases due to treatment [91], suggesting that MTF may lead, in time, to the selection of a favorable microbiota. Comparative evaluation of the fecal metagenome obtained from healthy and T2DM patients evidences dysbiosis in the gut microbiota. In particular, a lower abundance of *Firmicutes* and *Roseburia* and a higher abundance of *Bacteroides*, *Proteobacteria*, *Clostridium*, and *Lactobacillus* spp. has been observed in T2DM patients [87,88,90]. Treatment with MTF has been associated with decreased levels of *Clostridium* and *Eubacterium* and increased levels of *Escherichia*, *Shigella*, *Klebsiella*, *Salmonella*, and *Akkermansia muciniphila* [92,93]. Interestingly, treatment of T2DM patients with MTF increased the relative enrichment in SCFAs-producing bacteria. SCFAs, especially butyrate and propionate, are involved in improving several features of diabetes, including insulin resistance as well as inflammation, and in increasing GLP-1 secretion [71]. Moreover, a change in the microbiota composition in MTF-treated T2DM patients has been associated to modulation of bile acid metabolism [90]. In particular, it has been shown that MTF may increase the level of bile acids by decreasing the abundance of *Bacteroides fragilis* and of its bile salt hydrolase activity [94]. Another mechanism through which MTF might improve glucose metabolism through the modulation of gut microbiota is by maintaining the intestinal barrier functionality. Indeed, MTF treatment leads to a marked increase in the bacterium *Akkermansia muciniphila*, a mucin-degrading bacterium, which plays an important role in maintaining the integrity of the mucin layer, probably by improving the number of mucin-producing globet cells [67,95]. Therefore, it is plausible that the amelioration of glycemic control in MTF-treated T2DM patients may be mediated by alteration in the composition and improvement in the metabolic function of the microbiome.

Alteration of the gut bacterial composition by MTF also plays a role in reducing the risk of colon cancer in T2DM patients [96]. For instance, it has been shown that butyrate reduces the proliferation of cancerous colonocytes [97]. However, the protective effects of MTF in colon cancer seems to be due to its relative retention in the cells. Indeed, MTF has antiproliferative action in cancer cells with reduced expression of MATE2 [98]. Interestingly, metastatic colorectal cancer with reduced expression of MATE1 are more responsive to MTF [99]. These findings suggest that MTF needs to reach high concentrations in cells to exert protective effects from colorectal cancer, and that inhibition of Complex I activity may have a role in mediating MTF’s benefits.

### 2.2. The Effect of MTF on Liver Metabolism

The liver is the main organ involved in glucose metabolism management; hence, it is considered one of the principal targets of the MTF antidiabetic effect [20]. In particular, MTF determines a reduction of the hepatic glucose output, limiting gluconeogenesis, although the molecular mechanism associated with this effect still remains unclear [100,101]. Among the proposed mechanisms, the AMPK pathway activation due to the liver kinase B1 and the reduction of energy availability [100,102], the inhibition of glucagon-induced cAMP production [103], and the inhibition of mitochondrial shuttles [81] are indicated as the most likely. In addition, the inhibition of respiratory Complex I has been indicated as a possible mechanism [104], since its reduced activity should determine a decrement of the mitochondrial oxidative phosphorylation [19], which in turn causes a decrement of the ATP/AMP ratio, activating AMPK [21]. However, it is important to note that MTF inhibits the Complex I activity only over 1 mM [31], a concentration not reached in the hepatic tissue of subjects treated with therapeutic dose. Indeed, after gut adsorption, MTF is delivered to the liver through the portal vein, which displays a drug concentration between 40–70 μM or 10–40 μM before and after the liver uptake, respectively [7,105,106]. Other authors have observed that MTF could accumulate in the liver, within a range from 2- to 5-fold of the plasma concentration [17,19,106,107]. Moreover, MTF must enter into mitochondria to inhibit Complex I, but the mitochondrial inner membrane is impermeable, and no specific carrier for MTF has been identified yet [19]. Therefore, other mechanism could be involved in the liver regulation of gluconeogenesis flux—in some cases, independently from the of AMPK pathway activation, considering that the low MTF concentration does not determine a substantial lowering of intracellular ATP concentration [2,108].

It was proposed that AMPK signaling could be activated by the slight increment of the AMP level, dependent on the allosteric inhibition of fructose 1,6-bisphosphatase (FBP1) [109]. Alternative data suggest that MTF inhibits the mitochondrial FAD-dependent glycerol-3-phosphate dehydrogenase (mGPDH), one of the shuttles that transfers a reducing equivalent from the cytoplasm to mitochondria, determining a redox-dependent inhibition of gluconeogenesis and an increment of the lactate/pyruvate ratio [81,110]. This causes simultaneously an increment in the cytoplasmic NADH/NAD^+^ state and a reduction of the mitochondrial NADH/NAD^+^ ratio [111]. However, FBP1 inhibition implies a redox-independent inhibition of gluconeogenesis by oxidized and reduced substrates, while the mGPDH inhibition predicts a gluconeogenesis inhibition only by reduced substrates [112]. These discrepancies seem to have been resolved by the model proposed by Alshawi and Agius, in which the gluconeogenesis inhibition obtained with a treatment dose of MTF depends on a redox-independent mechanism [112]. These authors indicate the allosteric regulation of phosphofructokinase 1 (PFK1) by fructose 1,6-bisphosphate (F1,6P_2_) [100,113,114] and of FBP1 as a consequence of the reduction of glycerol 3-phosphate level [112].

MTF seems to display a positive effect on the liver not only in T2DM patients, but also in subjects with nonalcoholic fatty liver disease [115] and chronic hepatitis [116]. However, rare cases of hepatotoxicity have been described in fragile patients [117] treated with MTF. These cases seem to be associated with an increment in lactic acidosis, even if this is a very rare occurrence, representing less than three cases per 100,000 patients in one year [106]. Moreover, literature indicates that, in humans, MTF is accumulated principally at the glut level, suggesting that the small intestine is the prime site of MTF action [106] and possibly one in which inhibition of Complex I can actually occur. Nonetheless, sterologic measurements showed that mitochondria account for more than 20% of the hepatocyte volume [118]; therefore, it is doubtful that MTF can reach a concentration in the liver mitochondria sufficient for an inhibitory action, or even sufficient to exert any action.

### 2.3. The Effects of MTF on the Adipose Tissue

White adipose tissue, together with brown adipose tissue (WAT and BAT, respectively), composes the adipose organ, which constitutes as much as 20–25% of the body weight in healthy humans [119]. In particular, WAT acts as energy storage, is a major endocrine organ that produces several hormones (such as leptin, adiponectin, estrogen, resistin, and cytokines), and is a thermal insulator, contributing to the body temperature maintenance [120,121]. BAT is less represented in human adults, although it is the principal responsible of thermoregulation, due to the specific mitochondrial expression of uncoupling proteins (UCPs), which divert the proton gradient from ATP production to heat generation [122]. It is well known that increased BMI, obesity, and, therefore, the alteration of lean versus fat body mass, are one of the main risk factors for the development of insulin resistance and T2DM [123,124].

In WAT deposits, MTF reaches low levels, which, although in the micromolar range, are not comparable to that of other organs, such as the liver, the kidney, or the intestine [125]. This suggests that the classic mechanism of action proposed for MTF, i.e., mitochondrial Complex I inhibition, would not occur in this tissue.

According to different studies, the MTF-related reduction of body fat mass is due to the improvement in insulin sensitivity and, therefore, to the lower level of insulin released in the bloodstream [126]. Indeed, insulin action on lipid metabolism is anabolic, since it promotes fat storage synthesis and inhibits its release [127,128]. Moreover, in the literature, there is evidence that MTF has a direct effect on WAT, in particular stimulating adiponectin production [126,129,130]. MTF directly upregulates the adiponectin gene expression, both in vivo and in vitro, and stimulates adiponectin secretion from human subcutaneous adipose tissue in vitro [131]. Adiponectin is involved both in the regulation of glucose levels and fatty acid degradation, having a positive effect on T2DM [132]. Another study pointed out that MTF can induce oxidative stress in WAT, increasing the expression of uncoupling protein 2 (UCP2). UCP2 dissipates the mitochondrial proton gradient as heat and, at the same time, can minimize ROS levels [133]. Interestingly, UCP2 expression is one of the characteristic features of the conversion from WAT to BAT, a mechanism known as browning. Browning could be greatly beneficial for diabetic patients; however, another study has demonstrated that MTF administration in animal models can inhibit WAT browning [134]. Despite the mechanism of action on WAT and its correlation to browning still being unclear, MTF has an overall positive effect on the adipose organ.

Transplantation of embryonic BAT with IGF-1 supplementation in type 1 diabetic (T1D) adult mice was able to successfully correct T1D phenotypes, producing rapid and long-lasting normoglycemia, independent from insulin, at a 57% success rate [135], another outstanding example of the beneficial effects of activated BAT on diabetes. Investigating the distribution and accumulation of [^11^C]-MTF in different tissues in mice, an avid uptake of MTF in BAT deposits was observed. The MTF tissue-to-blood ratio in BAT was comparable to that of the liver during the first hour after intravenous administration. Interestingly, the cited study shows that after one hour from MTF administration, the liver uptake was slowly decreasing, while the absorption in BAT was still increasing [125]. This could suggest a MTF accumulation in BAT; however, a recent study demonstrated that micromolar MTF dosage can increase BAT mass and enhance mitochondrial biogenesis and thermogenesis [136], suggesting that administrating therapeutic dosages of MTF is not sufficient to reach the locoregional millimolar concentration necessary to inhibit mitochondrial Complex I. Moreover, if MTF reached high concentrations in BAT, leading to Complex I inactivation, it would be counterproductive for diabetic patients, as BAT induction and activation by cold exposure was proposed to be protective against obesity and diabetes, having a regulatory role in glucose homeostasis and insulin sensitivity, both in animal models and humans [137]. In conclusion, evidence suggests that MTF accumulation in BAT does not exceed the micromolar concentration and, therefore, is able to implement the mitochondrial functionality and the relative conversion of chemical energy in heating.

### 2.4. Effect of MTF on the Endothelium

Macro- and micro-angiopathy represents a major diabetes complication [138]. A healthy endothelium is crucial for vascular integrity, as it acts in maintaining a low level of oxidative stress and a relaxed vascular tone by balancing vasodilators (NO, prostacyclin I2, substance P) and vasoconstrictors (endothelin-1, angiotensin II) [139,140]. Hyperglycemia shifts glucose to the polyol pathways in the endothelial cells, generating advanced glycation end-products (AGEs) and activating protein kinase C (PKC) [141]. The resulting increased ROS production, mitochondrial dysfunction, and endothelial nitric oxide synthase (eNOS) impairment causes endothelial dysfunction (ED) and ultimately apoptosis [142].

Clinical studies show that MTF treatment ameliorates endothelial function [140,143]. MTF has a direct action against ED, likely due to improvement of insulin resistance and extra-glycemic effects, such as reduction of oxidative stress and of PKC and NADPH activation [144]. The beneficial effects on vasculature exerted by MTF comprise blood pressure reduction and endothelium-dependent relaxation (EDR) enhancement [145]. In detail, a study on Spontaneously Hypertensive Rats (SHR) shows that treatment with MTF appeared independent of its action in glycemic control and possibly dependent on the upregulation of the NO [145]. Moreover, the mechanisms involved in the vasoprotective effects of MTF may involve its activation of AMPK and of eNOS [146]. MTF restored endothelial function and improved NO bioavailability, lowering the oxidative stress in *Goto-Kakizaki* rats [147]. MTF inhibited the opening of the mitochondrial permeability transition pore (PTP) triggered by treatment with 30 mmol/L glucose in human microvascular endothelial cells (HMEC-1) as well as in primary endothelial cells from bovine aorta, preventing cell death [37]. The possibility that the beneficial effects of MTF on ED could also be mediated by its action on Complex I has been proposed [148]. However, it appears difficult to believe that the beneficial action of MTF on the endothelial aerobic metabolism could come from an inhibition of Complex I. Rather, it may derive from an indirect action on the mitochondrion, as a consequence of the activation of the NAD-dependent deacetylase Sirtuin 1, a key regulator of metabolism [148], or else from an activation of Complex I by the micromolar concentration of MTF conceivably present inside the endothelial cell in vivo.

### 2.5. Effects of MTF on the Heart

Diabetes is a well-recognized independent risk factor for the development of cardiovascular disease. In the last few years, a new player, i.e., MTF, emerged in the therapy of cardiovascular disease in diabetes. The UK Prospective Diabetes Study (UKPDS) was the first milestone trial to show that MTF reduces myocardial infarction risk [149]. Cardiomyopathy is one of the vascular complications associated with hyperglycemia in diabetes mellitus (American Heart Association, Cardiovascular disease and diabetes (2019); Available at: https://www.heart.org/en/health-topics/diabetes/diabetes-complications-and-risks/cardiovascular-disease--diabetes, accessed on 10 June 2021). Several studies have investigated the effects of MTF on the heart, with results mostly pointing to a cardioprotective role of MTF [150,151,152]. Notably, cardiomyocytes express relatively high levels of OCT3 [153].

A recent systematic review of randomized controlled trials showed that MTF reduces markers of heart failure in diabetic patients, improving myocardial oxygen consumption, although the molecular mechanisms of these effects are not completely understood, as there is no effect on left ventricular function [154]. Treatment with 100 mg MTF/kg/day for 12 months prevented heart failure in a rat model, and enhanced myocardial eNOS expression [155]. MTF pre-treatment in a pig Ischemia Reperfusion (I/R) model increased myocardial ATP levels after I/R [156]. Reduction of oxidative stress and myocyte apoptosis in animal models of I/R have been mainly related to AMPK activation and an increase in both eNOS activity and superoxide dismutase (SOD) expression [157]. However, MTF administered at the beginning of I/R in H9c2 cells improved mitochondrial respiration by activating the mitochondrial deacetylase protein sirtuin-3 (SIRT3), thus reducing cardiomyocytes oxidative stress and apoptosis; in fact, Sirtuins act in the overall regulation of metabolism [158]. Moreover, other authors propose that some effects of MTF on cardiomyocytes may be partially independent of AMPK and/or mitochondrial respiration [144], as observed in murine cardiomyocytes [159]. Other authors have described that in H9c2 cells maintained in high glucose pre-treatment with MTF before I/R restored mitochondrial membrane potential, and reduced mitochondrial permeability transition pore opening, activating Complexes I and III [160]. However, in the same cells, MTF was reported to inhibit Complex I activity, following I/R [161]. Interestingly, the treatment of mouse embryonic fibroblasts with MTF inhibited Complex I, whereas treatment of mice in vivo with MTF increased Complex I activity [144]. These opposite results may be due to the discrepancies between the millimolar concentrations of MTF used in vitro versus therapeutic blood concentrations (around 10 µM), and by the hydrophilicity of MTF and its low distribution volume [14,35]. In other words, low MTF concentrations activate Complex I and the relative ATP synthesis, while concentrations above 1 mM are inhibitory.

### 2.6. The Effect of MTF on Skeletal Muscle

Biodistribution of ^11^C-MTF, estimated through human radiation dosimetry by means of whole-body positron emission tomography (PET), revealed that MTF is primarily taken up by the kidney and liver but also by skeletal muscle, where it slowly accumulates with a reversible two-tissue-compartment kinetics [162]. In fact, the low-affinity, high-capacity MTF transporter OCT3 is expressed in human skeletal muscle [162]. In line with this, a recent report showed an increase in MTF content of human skeletal muscle over time (5 and 11 µM, respectively) after acute (1 day) or short-term (4 days) MTF treatment [163]. The same human study showed that very short-term MTF treatment does not affect skeletal muscle energy homeostasis and AMPK, although it causes enhanced perceived exertion and increased blood catecholamine levels [163]. By contrast, long-term (10 weeks) MTF treatment of diabetic subjects significantly increased AMPK α2 and decreased acetyl-CoA carboxylase-2 activity [164]. In fact, AMPK and its downstream signaling network is considered the main effector of MTF action on skeletal muscle. Notably, a consequence of AMPK activation by MTF is increased muscle glucose uptake and GLUT2 expression [165]. Moreover, it was observed that ATP and phosphocreatine concentrations were lower after MTF treatment [164], suggesting a depressed energy status, consistent with the hypothesis of MTF accumulation in skeletal muscle and the generally inhibitory effect of MTF on muscle mitochondrial function reported in both diabetic humans and animal models of diabetes [166,167].

Oral treatment of control and Zucker diabetic fatty rats with MTF for 2 weeks demonstrated that MTF significantly impairs the oxidative capacity of skeletal muscle in vivo and mitochondrial function ex vivo at high dosages (100–300 mg/kg/day) [166]. In humans, MTF was reported to partially blunt some beneficial adaptations to exercise. In a double-blind randomized study on a cohort of older human subjects at risk for T2DM treated with MTF at clinical doses for 2 weeks, MTF inhibited the improvement in mitochondrial respiration and insulin sensitivity of the skeletal muscle that should have been achieved after 12 weeks of aerobic exercise training. Such antagonism of MTF for the exercise adaptations and the generally depressed energy status of skeletal muscle during long-term treatment have been related to the inhibition of muscle mitochondrial respiration and to the action of MTF on Complex I [167]. However, biopsies of skeletal muscle from T2DM patients treated with MTF did not show suppressed Complex I activity [32]. Furthermore, the self-selected exercise intensity was not reduced by MTF in healthy males, even though MTF increased the rate of perceived exertion during an exercise bout with fixed intensity [168]. Moreover, MTF administration seems to activate a mechanism directly related to the hormetic response through the redox state modulation [169].

MTF interference with mitochondria, if any, appears protective of the heart and endothelium but less protective of muscle. Such a discrepancy may be due to the different substrate preferences: cardiac function mostly relies on the oxidative metabolism of fatty acids inside the mitochondria [170], while the skeletal muscle essentially relies on glucose [165].

## 3. Conclusions

**Figure 2:**
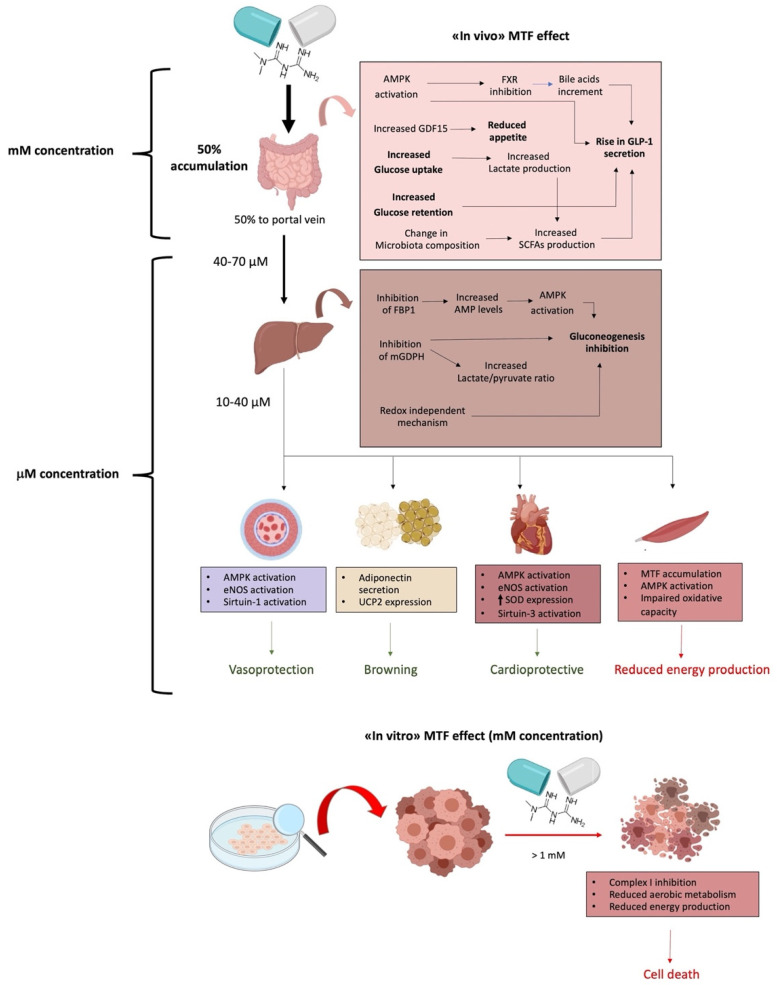
Schematic representation of the effects of micromolar (μM) or millimolar (mM) metformin (MTF) concentrations. The upper part of the figure represents the effect of MTF at the therapeutic concentration (in vivo—millimolar in liver and micromolar in the rest of body). The lower part of the figure depicts the effect of MTF at doses usually used in vitro experiments (millimolar).

The use of MTF progressively expanded from diabetes to obesity, liver disease, and, finally, cancer therapy. Interestingly, MTF is an example of hormesis, the dose–response phenomenon characterized by a low dose stimulation and high dose inhibition, resulting in an adaptive response to cellular metabolism variations. In detail, millimolar MTF concentrations, used in vitro, caused inhibition of the mitochondrial Complex I, a decrement in chemical energy production, and the consequent activation of the AMPK pathway (Figure 2). Conversely, micromolar MTF concentrations, which correspond to the therapeutic dosage, activated the mitochondrial aerobic metabolism, improving the cellular energy status. It appears that this peculiarity of the mode of action of MTF should be considered when the observations from in vitro studies are translated into in vivo treatments, bearing in mind the different tissue concentrations/accumulation of MTF. Based on the concept of hormesis, this review provides a comprehensive justification of the apparently opposite effects of MTF on single organs, in face of its overall beneficial effect. Currently, the MTF dose (generally, 500–2500 mg/die) depends mainly on the goal of glycated hemoglobin to be achieved. Indeed, it is difficult to estimate the actual concentration of MTF in the various tissues, as it may also differ in a single individual due to the clinical characteristics of each patient. The concept of hormesis can be useful to fully understand the effects of MTF in each patient, thus obtaining effective tailoring of MTF therapy, not only in diabetes.

## Data Availability

Not applicable.
